# Update on clinical heart failure trials

**DOI:** 10.1007/s00392-025-02784-4

**Published:** 2025-11-17

**Authors:** Julian Hoevelmann, Mert Tokcan, Saarraaken Kulenthiran, Amr Abdin, Charle Viljoen, Michael Böhm

**Affiliations:** 1 Department of Internal Medicine III - Cardiology, Angiology and Intensive Care Medicine, Saarland University Hospital, Saarland University, Homburg , Germany; 2 HOMICAREM (HOMburg Institute of CArdioREnalMetabolic Medicine), Medical Faculty, Saarland University, Saarbrücken, Germany; 3 Cape Heart Institute, Faculty of Health Sciences, University of Cape Town, Cape Town, South Africa

**Keywords:** Heart failure, Inflammation, Heart failure with preserved ejection fraction, Heart failure with reduced ejection fraction

## Abstract

Heart failure (HF) remains a major global health burden, with rising prevalence and significant morbidity and mortality despite advances in diagnosis and management. Over the past years, the therapeutic landscape of HF has evolved substantially, driven by a growing number of pivotal clinical trials addressing not only heart failure with reduced ejection fraction (HFrEF), but also heart failure with mildly reduced (HFmrEF) and preserved ejection fraction (HFpEF). The following review provides a comprehensive update on key clinical trials published or presented in the recent past such as DIGIT-HF, VICTORIA and VICTOR, STRONG-HF, SUMMIT or STEP-HFpEF trials, highlighting novel pharmacological therapies, patient phenotyping, and treatment of co-morbidities. Special attention is given to trials with practice changing implications and those shaping future guidelines.

**Graphical Abstract:**

Central illustration: *ACEi* angiotensin-converting enzyme inhibitor, *ARB* angiotensin receptor blocker, *ARNI* angiotensin receptor–neprilysin inhibitor, *CV* cardiovascular, *GDMT* guideline-directed medical therapy, *GLP-1* glucagon-like peptide-1, *HFmrEF* heart failure with mildly reduced ejection fraction, *HFpEF* heart failure with preserved ejection fraction, *HFrEF* heart failure with reduced ejection fraction, *HHF* hospitalization for heart failure, *LVEF* left ventricular ejection fraction, *MRA* mineralocorticoid receptor antagonist, *SGLT2i* sodium-glucose cotransporter 2 inhibitor, *uNa⁺* urinary sodium.

**Figure:**
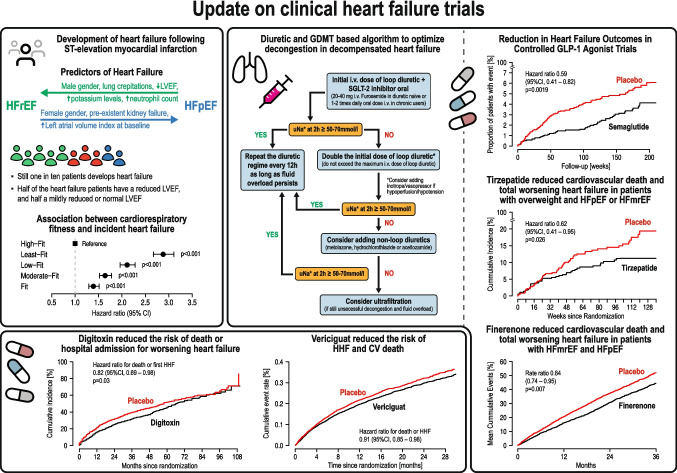

## Introduction

Heart failure (HF) remains a major global health burden, with rising prevalence and significant morbidity and mortality despite advances in diagnosis and management [1]. Over the past years, the therapeutic landscape of HF has evolved substantially, driven by a growing number of pivotal clinical trials addressing not only heart failure with reduced ejection fraction (HFrEF) but also heart failure with mildly reduced ejection fraction (HFmrEF) and preserved ejection fraction (HFpEF). The following review provides a comprehensive update on key clinical trials published or presented in the recent past such as DIGIT-HF, VICTORIA and VICTOR, STRONG-HF, SUMMIT, or STEP-HFpEF trials, highlighting novel pharmacological therapies, patient phenotyping, and treatment of co-morbidities. Special attention is given to trials with practice-changing implications and those shaping future guidelines (Central illustration, Table 1).

**Table 1:** Summary of important recent studies with practice-changing implications for the management of heart failure

Topic	Author (year)	Study design	Key findings
Incident heart failure	Lenselink et al. (2024)	Observational study	During a median follow-up period of 3.7 years after STEMI, one in ten patients still developed heart failure, predominantly HFrEF. The development of HFrEF was predicted by male sex, lung crepitations, increased potassium levels, an increased neutrophil count, a reduced LVEF at baseline, and for HFpEF by female sex, pre-existing kidney failure, and a higher left atrial volume index at baseline
	Kokkinos et al. (2024)	Observational cohort from the Exercise Testing and Health Outcomes Study (ETHOS)	In U.S. veterans, where CRF was objectively assessed using a standardized exercise treadmill test, adjusted HFpEF risk decreased as CRF increased, independently of comorbidities. Changes in CRF were proportional to changes in HFpEF risk, suggesting that HFpEF risk is modulated by CRF
Treatment	Armstrong et al. (2020)	Multicenter, double-blind, randomized, placebo-controlled trial	In patients with recent worsening HFrEF despite GDMT, vericiguat significantly reduced the composite outcome of cardiovascular death or HF hospitalization compared with placebo. The greatest benefit was observed among patients with moderately elevated NT-proBNP levels
	Veltmann et al. (2024)	Multicenter, prospective observational study	In patients with de novo HFrEF (≤ 4 months, LVEF ≤ 35%), a substantial proportion achieved recovery of LVEF within 3–12 months of optimized GDMT, often exceeding thresholds for primary preventive ICD implantation. These findings underscore the importance of early GDMT optimization and careful reassessment of ICD indication over time
	Biegus et al. (2024)	Literature review	A diuretic-based treatment approach reduces volume overload and achieves rapid decongestion. However, it is accompanied by neurohormonal activation, which can result in counter-regulatory sodium and water retention. The GDMT-based approach targets the neurohormonal pathways that drive fluid retention. By mitigating these mechanisms, GDMT may enable more effective and sustained decongestion. This dual-action model could explain the favorable outcomes observed during acute heart failure decompensation
	Kosiborod et al. (2024)	Pooled analysis of the SELECT, FLOW, STEP-HFpEF, and STEP-HFpEF DM randomized trials	Combined data from these randomized controlled studies demonstrated a 41% relative risk reduction in cardiovascular death and worsening heart failure with GLP-1 receptor agonist therapy in patients with mildly reduced or preserved ejection fraction
	Packer et al. (2024)	Double-blind, randomized, placebo-controlled trial	The dual GLP-1/GIP agonist resulted in a 38% reduction in cardiovascular death and heart failure hospitalizations and improved health status in patients with obesity and HFpEF
	Solomon et al. (2024)	Double-blind, randomized, placebo-controlled trial	In patients with HFmrEF or HFpEF, finerenone significantly reduced the rate of a composite of total worsening heart failure events and death from cardiovascular causes by 16%
	Berg et al. (2025)	Multicenter, double-blind, randomized, placebo-controlled trial	In patients hospitalized for HF, initiation of dapagliflozin during hospitalization did not significantly reduce CV death or worsening HF at 2 months. However, a pre-specified meta-analysis of three trials (EMPULSE, DAPA ACT HF-TIMI 68, SOLOIST-WHF) showed significant reductions in worsening HF and all-cause mortality with in-hospital initiation of SGLT2 inhibitors
	Bavendiek et al. (2025)	Multicenter, double-blind, randomized, placebo-controlled trial	In patients with symptomatic HFrEF on optimized GDMT, the addition of digitoxin significantly reduced the composite of all-cause mortality or hospitalization for worsening HF compared with placebo
	Butler et al. (2025)	Multicenter, double-blind, randomized, placebo-controlled trial	In stable ambulatory patients with HFrEF and elevated natriuretic peptides but without recent decompensation, the VICTOR trial did not show a significant reduction in the primary composite endpoint with vericiguat, although reductions in CV and all-cause mortality were observed
Decongestion	Herrmann et al. (2025)	Multicenter, randomized, open-label clinical trial	Liberal vs restricted fluid intake does not negatively impact quality of life and may reduce thirst distress compared to fluid restriction
Comorbidities	Haehling et al. (2024)	Double-blind, randomized, placebo-controlled trial	In patients with HFpEF and iron deficiency, intravenous iron administration improved 6MWT distance by approximately 20–40 m over 32 weeks. While this is the first controlled trial of intravenous iron in HFpEF patients, the trial lacked sufficient power to identify or refute effects on symptoms or quality of life
	Anker et al. (2025)	Multicenter, randomized clinical trial	Ferric carboxymaltose did not significantly reduce the time to first heart failure hospitalization or cardiovascular death in patients with heart failure

*HFrEF* heart failure with reduced ejection fraction, *HFpEF* heart failure with preserved ejection fraction, *CRF* cardiorespiratory fitness, *GDMT* guideline-directed medical therapy, *NT-proBNP* N-terminal pro-B-type natriuretic peptide, *GLP-1* glucagon-like peptide-1, *GIP* glucose-dependent insulinotropic polypeptide, *HFmrEF* heart failure with mildly reduced ejection fraction, *6MWT* six-minute walk test, *MRA* mineralocorticoid receptor antagonist

## Risk factors

The primary risk factors for the development of chronic HF include coronary artery disease and traditional cardiovascular (CV) risk factors such as arterial hypertension, diabetes mellitus, and obesity—particularly in cases of HFpEF. Additional contributors include a history of myocarditis or chronic pressure and volume overload. These factors are further aggravated in patients with an underlying genetic predisposition [2]. A research group in the Netherlands conducted a 3.7-year follow-up study on a cohort of patients with ST-elevation myocardial infarction (STEMI) to evaluate the development of HFpEF or HFrEF [3]. During follow-up, 52.1% of patients developed HFrEF, 18.5% developed HFpEF, and 29.4% developed HFmrEF. Predictors for the development of HFrEF included male sex, signs of congestion such as pulmonary crackles during the acute infarction, impaired systolic function, and elevated leukocyte counts. In contrast, predictors for HFpEF were female sex, pre-existing renal insufficiency, and increased left atrial volume at the time of infarction. The incidence of post-myocardial infarction HF was 10% [3].

A similar study investigated the predictive value of renal function for the development of HF, using data from the Prevention of Renal and Vascular Endstage Disease (PREVEND) study. The findings demonstrated that patients with a CV risk profile (particularly those with elevated baseline serum creatinine levels and increased urinary albumin excretion) were more likely to develop new-onset HF [4]. As such, any patient presenting with microalbuminuria and impaired renal function should be considered at high risk for developing HF. In these patients, guideline-recommended preventive pharmacological interventions, such as the use of sodium-glucose cotransporter 2 (SGLT2) inhibitors and the non-steroidal mineralocorticoid receptor antagonist (nsMRA) finerenone, should be prescribed [2].

Furthermore, a larger study assessed the association between cardiorespiratory fitness and the risk of developing HFpEF. The results showed that patients in the lowest fitness quartile had nearly a threefold increased risk of HFpEF [5]. Within this subgroup, additional predictors, such as chronic kidney disease (CKD), advanced age, atrial fibrillation, obstructive sleep apnea, and diabetes mellitus, were associated with an incremental risk increase of 30–114%.

## Worsening, decompensated, and acute (“de novo”) heart failure

Worsening heart failure (WHF) is defined as a deterioration in symptoms, clinically characterized by changes in biomarkers, the occurrence of events such as hospitalization or emergency presentation, or the need for escalation of intravenous diuretic therapy, all of which significantly impact long-term prognosis and quality of life. These definitions have been clearly outlined and standardized in a recent consensus document [6]. Acute “de novo” HF refers to the first clinical manifestation of HF and is typically associated with a poorer prognosis and a lower intensity of therapy prior to decompensation. In contrast, the treatment of acute-on-chronic heart failure primarily focuses on symptomatic relief (e.g., decongestion and diuretic therapy) as well as on improving long-term outcomes using neurohormonal antagonists and SGLT2 inhibitors. These therapies aim to prevent recurrent decompensation and the transition into a so-called "frequent-flyer" phenotype [6]. Ultimately, congestion leading to acute deterioration is driven in part by neurohormonal activation. Therefore, the method of decongestion and the early initiation of guideline-directed medical therapy (GDMT) not only affect recurrence rates but also modulate neurohormonal activity during both decompensation and the recompensation phases [6]. Beyond the four established pillars of GDMT for HFrEF, the international, double-blind, placebo-controlled DIGIT-HF trial has recently provided new therapeutic perspectives. The study included 1212 patients with symptomatic HFrEF receiving a well-implemented background HF therapy that were randomized to receive the cardiac glycoside digitoxin or placebo. The addition of digitoxin significantly reduced the risk of the composite endpoint of all-cause mortality or hospitalization for WHF compared with placebo. This translated into an absolute risk reduction (ARR) of 4.6% and a number needed to treat (NNT) of 22, suggesting that digitoxin may represent a valuable adjunctive option in the contemporary management of HFrEF [7].

In addition, the oral soluble guanylate cyclase stimulator vericiguat has been evaluated in two large phase III trials across different spectra of HFrEF. In the VICTORIA trial, which enrolled more than 5000 patients with recent WHF despite optimized therapy, vericiguat significantly reduced the composite endpoint of cardiovascular death or HF hospitalization compared with placebo, with the greatest benefit observed in patients with moderately elevated N-terminal pro-brain natriuretic peptide (NT-proBNP) [8]. In contrast, the VICTOR trial investigated over 6000 more stable ambulatory patients with HFrEF and elevated natriuretic peptides but without recent decompensation for HF [9]. Here, vericiguat did not significantly lower the primary composite endpoint, though reductions in cardiovascular and all-cause mortality were observed [9]. A pre-specified, pooled individual participant-level analysis of the two trials, including more than 11,000 patients, demonstrated a consistent reduction of the risk of hospitalization for HF and CV death in patients with HFrEF across a broad range of clinical severity, supporting its role as an adjunctive therapy for selected patients with HFrEF [10].

## Treatment

### Decongestion

Sequential nephron blockade is recommended for the management of decongestion in acute heart failure (AHF). This strategy involves the combination of loop diuretics, which act on the ascending limb of the loop of Henle, with distally acting agents such as thiazide diuretics and MRAs, as well as agents acting on the proximal tubule such as SGLT2 inhibitors or carbonic anhydrase inhibitors like acetazolamide [11]. A recent study evaluated the efficacy of the SGLT2 inhibitor dapagliflozin compared to the thiazide-like diuretic metolazone in patients with relative loop diuretic resistance. The results showed that dapagliflozin had a similar efficacy to metolazone, which was previously used as a thiazide-like agent in clinical practice in Germany [12]. Similarly, the ADVOR trial demonstrated that the addition of acetazolamide to intravenous loop diuretics enhances decongestion, as reflected by weight loss and reduction in congestion symptoms, in patients with decompensated AHF [13]. Further evidence suggests that effective decongestion is achievable even in patients with impaired renal function. In these cases, the degree of decongestion was associated with higher baseline bicarbonate levels, supporting the notion that natriuresis induced by carbonic anhydrase inhibitors is more pronounced at elevated bicarbonate concentrations [14].

The STRONG-HF trial investigated the early initiation of intensive GDMT in conjunction with aggressive diuretic therapy initiated prior to hospital discharge [15]. A subsequent secondary analysis showed that, despite ongoing diuretic treatment during decompensation, there was a significant elevation of NT-proBNP, reflecting increased wall stress. This neurohormonal activation was significantly attenuated with the concurrent administration of GDMT. These findings suggest that early reduction of wall tension and filling pressure, along with the simultaneous initiation of diuretic therapy and prognostically beneficial GDMT may help reduce hospital readmissions. In STRONG-HF, this approach led to an absolute reduction of 8.4% in all-cause mortality and hospitalizations within 6 months [16].

Additionally, glucagon-like peptide-1 (GLP-1) receptor agonists have shown promising effects in the STEP-HFpEF trials, both in patients with and without diabetes mellitus. These agents were associated with reduced hospitalization rates, improved functional capacity, and enhanced quality of life [17, 18]. A secondary analysis revealed that this therapy also allowed for a reduction in diuretic dosage, likely driven by the observed weight loss of up to 15% and potentially due to direct cardiac and renal effects. Similar outcome improvements have been reported with SGLT2 inhibitors, angiotensin receptor-neprilysin inhibitor (ARNi), and MRA (spironolactone) in patients with HFmrEF or HFpEF [19].

In addition, an important trial addressed the issue of fluid restrictions in patients with chronic HF. The multicenter, open-label FRESH-UP trial randomized patients with HF to a liberal versus restricted (up to 1500 mL daily) fluid intake. The primary endpoint was health status at 3 months, evaluated using the Kansas City Cardiomyopathy Questionnaire Overall Summary Score (KCCQ-OSS). Secondary outcomes encompassed measures of thirst distress and safety events. At 3 months, no significant difference in the primary outcome was observed, while thirst distress was significantly lower in the group of patients with liberal intake. This suggests that a liberal fluid intake does not negatively impact quality of life and may reduce thirst distress compared to fluid restriction [20].

Collectively, these findings support the indispensable role of decongestive therapy with diuretics while highlighting the importance of early initiation of neurohormonal therapies that modulate the renin–angiotensin–aldosterone system and sympathetic nervous activity. This represents a paradigm shift—from a diuretic-centric to a combined diuretic and GDMT-centric approach in the treatment of acutely decompensated heart failure (ADHF) [21].

### Inotropic support

Positive inotropic agents are recommended for the treatment of AHF in the presence of hypotension, signs of hypoperfusion, and elevated filling pressures, particularly when these cannot be adequately managed with diuretics or vasodilators due to low blood pressure [2]. In patients with terminal HF, inotropic support may improve quality of life, though without evidence of a survival benefit [2]. Most data associating inotropes with increased mortality are based on studies involving dobutamine or milrinone. The calcium sensitizer levosimendan has shown promising results in pilot studies and one controlled trial, suggesting similar or potentially superior efficacy compared with conventional inotropes in cases of severe heart failure. Mechanistically, a lack of direct β-adrenergic receptor stimulation combined with an enhanced calcium sensitivity of contractile proteins and inhibition of phosphodiesterase may confer advantages in certain clinical scenarios [22]. Until recently, there were no randomized controlled trials evaluating levosimendan in patients with terminal HF following hospital discharge, aiming to prevent recurrent hospitalization. The LeoDOR trial [23] addressed this gap by comparing levosimendan with placebo in this population. However, results showed higher rates of decompensation and an overall unfavorable risk–benefit profile for levosimendan, thereby failing to demonstrate a clear advantage [23].

A consensus document from the Heart Failure Association (HFA) of the European Society of Cardiology (ESC) has delineated the remaining indications for inotropic therapy in advanced HF. Inotropic support may be considered in select cases, including irreversible myocardial dysfunction in end-stage heart failure, contraindications for left ventricular assist devices (LVAD) or heart transplantation, severe diuretic resistance, hemodynamic optimization for symptomatic relief, and bridging therapy while awaiting heart transplantation or LVAD implantation [24].

### Initiation during hospitalization

Current HF guidelines recommend initiating GDMT during hospitalization following ADHF [2]. This recommendation is primarily supported by data from the STRONG-HF trial [15], which demonstrated that early initiation of comprehensive GDMT combined with intensive diuretic therapy significantly reduced rehospitalizations and mortality.

The EMPULSE trial specifically investigated in-hospital initiation of the SGLT2 inhibitor empagliflozin in patients with AHF, including those with HFpEF [25]. A post hoc analysis confirmed consistent benefit across the full spectrum of ejection fractions using a hierarchical win ratio approach, suggesting a homogeneous risk reduction with empagliflozin [26]. Similarly, the Dapagliflozin Effect on Cardiovascular Events in Acute Heart Failure – Thrombolysis in Myocardial Infarction 68 (DAPA ACT HF-TIMI 68) trial investigated the efficacy and safety of in-hospital initiation of the SGLT2 inhibitor dapagliflozin in patients hospitalized for HF. In this trial, in-hospital initiation of dapagliflozin did not significantly reduce the risk of CV death or worsening HF through 2 months. However, a pre-specified meta-analysis of three trials (EMPULSE, DAPA ACT HF-TIMI 68, and SOLOIST-WHF [27]) evaluating in-hospital initiation of SGLT2 inhibitors demonstrated a significant reduction in WHF (HR 0.71; 95% CI 0.54–0.93) and of all-cause death (HR 0.57; 95% CI 0.41–0.80) [28]. The PREMIER trial evaluated sacubitril/valsartan across different ejection fraction ranges in AHF. While positive effects were seen overall, the benefit diminished in patients with ejection fractions above 50%, suggesting that SGLT2 inhibitors may exert greater efficacy in patients with HFpEF than ARNI therapy in this setting [29]. Real-world data from U.S. hospital cohorts corroborate these findings, showing favorable outcomes with SGLT2 inhibitors in patients with ejection fractions > 40% [30, 31].

A consensus statement by the HFA of the ESC provides a structured treatment guide for both in-hospital and early post-discharge management following decompensation. It emphasizes initiating the full spectrum of GDMT during hospitalization and titrating to the maximum tolerated dose shortly after discharge (within ~ 2 weeks), regardless of ejection fraction [32].

In light of the recent advancements in HF treatment, the HF-OPT trial has provided important insights into the natural course of newly diagnosed HFrEF under contemporary GDMT. This prospective trial enrolled 598 patients with de novo HFrEF (≤ 4 months, LVEF ≤ 35%) and systematically evaluated the trajectory of LV function while deferring implantation of a prophylactic implantable cardioverter-defibrillator (ICD). The study demonstrated that a considerable proportion of patients achieved meaningful recovery of LVEF within 3 to 12 months of optimized medical therapy, often exceeding thresholds for primary preventive ICD implantation. These findings underscore the value of prioritizing early and intensive optimization of GDMT, while carefully reassessing the indication for device implantation in this patient population.

### Finerenone

MRAs are indicated for patients with HFrEF and post-myocardial infarction HF [2]. Finerenone, a novel non-steroidal MRA, was evaluated in the FINEARTS-HF trial, which enrolled patients with HFmrEF and HFpEF (LVEF > 40%). The study demonstrated a 16% relative risk reduction in the composite primary endpoint of cardiovascular death or HF hospitalization/emergency visits, with an ARR of 3.3% [33]. Secondary endpoints showed an 18% reduction in all HF hospitalizations, while the reduction in CV mortality (7%) did not reach statistical significance. A total of 6001 patients were randomized in the trial [33]. Patients enrolled shortly after or during an acute decompensation phase had a higher baseline risk [34] and showed a smaller absolute risk reduction [35]. Nevertheless, the therapeutic effect of finerenone emerged rapidly, i.e., within 28 days of initiation [36]. These findings support early initiation of finerenone following a confirmed diagnosis of HFmrEF or HFpEF, especially after a recent decompensation for HF, to reduce the risk of subsequent events. Life expectancy gains without cardiovascular events were notable: 3.1 years in patients > 55 years, 3.0 years in those > 65 years, and 0.6 years in patients > 75 years [37]. Hyperkalemia occurred at twice the rate seen in the placebo group, although hypokalemia was reduced by 50% [33]. Hyperkalemia was more common in patients with eGFR < 60 mL/min/1.73 m^2^, although the relative increase in incidence was consistent across kidney function strata [38]. Serum potassium levels increased by approximately 0.2–0.3 mmol/L. Direct comparisons with other MRA trials investigating eplerenone or spironolactone are scientifically limited due to differing renal functions across studies. However, the risk of hyperkalemia appears to be lower with finerenone [39]. Risk reduction benefits extended across the full range of serum potassium concentrations [38]. A recent meta-analysis of all MRA trials spanning the full range of ejection fractions showed comparable effects for spironolactone, eplerenone, and finerenone. However, a significant interaction was observed, with greater efficacy in HFrEF and smaller effects in HFmrEF and HFpEF [40].

### GLP-1 receptor agonists and tirzepatide

Patients with obesity exhibit a high incidence of HFpEF as well as HFmrEF [2]. GLP-1 receptor agonists reduce CV risk in individuals with overweight and type 2 diabetes mellitus, as demonstrated in the SELECT trial, which included 16,604 high-risk patients [41]. Moreover, liraglutide [42] and semaglutide [43] were shown to attenuate the decline in glomerular filtration rate (GFR) and reduce renal endpoints.

In patients with HFpEF or HFmrEF and elevated body mass index (BMI), the GLP-1 receptor agonist semaglutide improved health-related quality of life, as measured by the KCCQ, and increased six-minute walk distance, both in patients with and without diabetes mellitus [17, 18]. In both populations, improvements in these patient-reported outcomes were associated with reductions in CRP and IL-1 levels [17, 18]. Furthermore, enhanced functional capacity and decreased systemic inflammation were correlated with reductions in body weight [44]. These findings indicate that semaglutide produces a robust improvement in patient-centered outcomes. A meta-analysis of the SELECT, FLOW, and STEP-HFpEF trials revealed reductions in HF hospitalizations and CV mortality [45]. This increased statistical power supports the hypothesis that GLP-1 receptor agonists may have prognostic benefits in patients with HFpEF and HFmrEF. However, it is worth noting that individuals with low BMI were excluded from many of these trials. Overall, these results suggest that weight reduction may play a pivotal role in mitigating comorbidities and improving patient-centered outcomes in HF. This is further supported by recent data showing that incident HF was even more significantly reduced following bariatric-metabolic surgery than with GLP-1 receptor agonist therapy [46]. Importantly, two recent studies found no evidence of increased suicidality associated with GLP-1 receptor agonists [47, 48]. Current debate includes concerns regarding loss of lean muscle mass, which has been reported to account for up to 39% of total weight reduction in semaglutide trials [49, 50]. This effect appears to be less pronounced with the dual glucose-dependent insulinotropic polypeptide (GIP) receptor and GLP-1 receptor agonist tirzepatide [49]. A potential direct effect on myocardial mass has also been hypothesized. Experimental studies in animal models demonstrated reductions in myocardial mass, even in the absence of pre-existing disease [51]. It remains unclear whether echocardiographic or cardiac MRI findings reflect true myocardial tissue loss or simply reductions in interstitial fat, which current imaging modalities cannot reliably quantify. Additional obesity-related improvements, such as relief of knee joint pain from osteoarthritis and reductions in obstructive sleep apnea, have also been reported [52, 53]. Given the strong association between weight loss and improved HF outcomes, it is notable that dual agonism of the GLP-1 and GIP receptors yields more substantial weight reduction than GLP-1 receptor agonism alone [54].

In the recently presented SUMMIT trial [55], tirzepatide reduced the composite endpoint of cardiovascular death, HF hospitalization, or urgent HF visits by 38% [55]. A placebo-controlled 11.6% weight reduction after 52 weeks was associated with significant improvements in six-minute walk distance. Similar to GLP-1 trials, there was a reduction in CRP and troponin levels, with no significant change in NT-proBNP concentrations [56]. Additionally, tirzepatide led to a reduction in microalbuminuria, although renal function as assessed by eGFR remained unaffected due to the limited sample size [56].

### Novel anti-inflammatory approaches

The activation of inflammatory pathways in HF has long been recognized, particularly in the context of acute and advanced disease stages [57, 58]. Inflammation may contribute to endothelial barrier dysfunction, potentially playing a role in the pathogenesis of pulmonary edema [59].

The CORTA-HF trial investigated patients with AHF exhibiting both inflammatory activation and elevated NT-proBNP levels. Participants were randomized 1:1 to receive either 40 mg prednisolone or placebo. The intervention group demonstrated a significant reduction in inflammatory markers, specifically C-reactive protein (CRP) and interleukin-6 (IL-6). Moreover, improvements in quality of life were observed by day 30, along with a reduction in CV events within the first 90 days. Although the study was exploratory and not powered for definitive clinical endpoints, the findings are promising and warrant further investigation. Notably, aside from mild hyperglycemia, there were no significant increases in sodium retention or hypertensive events [60].

Similar findings emerged from the COLICA trial, in which colchicine was administered at a maintenance dose of 0.5 mg/day following a loading dose to patients with acute heart failure [61]. This intervention resulted in a marked reduction in CRP and IL-6 levels; however, NT-proBNP concentrations did not decline significantly. With a sample size of 274 patients, the study lacked statistical power to assess clinical efficacy [62]. Therefore, colchicine remains primarily indicated for patients with coronary artery disease [63], pending results from larger trials evaluating its role in heart failure populations.

## Comorbidities

### Chronic kidney disease (CKD)

CKD increases the incidence and worsens cardiovascular outcomes in patients with heart failure [64]. Most pharmacological interventions remain effective across the full spectrum of renal impairment stages [65]. A key question, however, is whether therapies such as ARNI or MRA should be withheld or discontinued when GFR falls below 30 mL/min/1.73 m^2^. Recent evidence suggests that patients progressing to end-stage renal disease (KDIGO stage 5) still derive CV benefit from continued MRA therapy [66]. Moreover, studies indicate that the effect of MRAs may even be more pronounced in HF patients with an eGFR < 30 mL/min/1.73 m^2^, though this observation did not reach statistical significance [67–69]. The primary concern regarding continued MRA use in renal impairment is the risk of hyperkalemia. In this context, co-administration of SGLT2 inhibitors or ARNI has been shown to mitigate MRA-associated hyperkalemia [67, 69]. Thus, comorbidities may not only be additive in combination therapy but may also confer reciprocal therapeutic benefits.

### Low blood pressure

One of the main barriers to the implementation of GDMT in HF is low blood pressure, as many guideline-recommended drugs exert hypotensive effects. In clinical trials, patients with low baseline blood pressure exhibited higher event rates in placebo arms, while the treatment benefit of SGLT2 inhibitors was preserved [70, 71]. A recent analysis from the Swedish Myocardial Infarction Registry confirmed the association between low blood pressure and higher event rates, while also demonstrating that the relative risk reduction from GDMT was maintained even at higher drug doses [72]. These data support the continued and titrated use of GDMT in hypotensive patients, tailored to individual tolerability and adverse effects [70].

### Iron deficiency

Iron deficiency affects approximately 50% of patients with chronic HF [73]. Intravenous iron supplementation improves quality of life and reduces hospitalization rates in patients with HFmrEF and HFrEF, supporting its recommendation in current guidelines for quality-of-life improvement [74–76]. Data in patients with HFpEF have been lacking. However, the recently published FAIR-HFpEF trial demonstrated that patients with transferrin saturation (TSAT) < 20% experienced a placebo-controlled increase in six-minute walk distance of approximately 45%, suggesting a beneficial effect in this population [77]. In contrast, the FAIR-HF2 trial enrolled 1105 patients with chronic heart failure (defined as having a LVEF of ≤ 45%) and iron deficiency (serum ferritin level < 100 ng/mL; or if TSAT was < 20%, a serum ferritin level between 100 and 299 ng/mL). In this trial, ferric carboxymaltose did not significantly reduce the primary endpoint of the time to CV death or first HF hospitalization in this population [78]. It is important to emphasize that TSAT < 20%, rather than ferritin concentration, should be used to define iron deficiency. TSAT is more strongly associated with adverse prognosis, whereas elevated ferritin may reflect inflammation. This diagnostic paradigm shift represents a major advancement in the detection and management of iron deficiency in HF [79].

### Infections

The COVID-19 pandemic has caused significant mortality, particularly in patients with pre-existing cardiovascular disease. In the DELIVER trial (dapagliflozin in HFpEF), a sub-analysis examined the impact of pulmonary infections on prognosis. Results showed a 4–sixfold increase in mortality among patients with HFmrEF and HFpEF following COVID-19 infection [80]. Similarly, in the EMPEROR-Preserved trial, urinary tract infections were associated with a threefold increase in mortality [81]. Interestingly, empagliflozin therapy was associated with a 17% reduction in incident respiratory infections. Influenza vaccination was associated with a 37% reduction in mortality and HF hospitalization [82], and the protective effect was more pronounced in patients who reported post-vaccination side effects [83]. These outcomes align with, yet surpass in clarity, the currently understated guideline recommendation (class IIa, level of evidence B) for influenza and pneumococcal vaccination. In summary, patients with HF, especially those at elevated risk, should be vaccinated according to current recommendations [2].

## Conclusion

HF represents a growing global challenge, with recent trials expanding evidence-based therapies across the full spectrum of ejection fractions. Key advances include early in-hospital initiation of GDMT, broader use of SGLT2 inhibitors, and emerging therapies such as finerenone, vericiguat, digitoxin, and GLP-1 and dual GLP-1/GIP receptor agonists, alongside refined strategies for decongestion, and management of comorbidities. Novel anti-inflammatory approaches and precision phenotyping of patients may further optimize outcomes.
